# Changes in the Phylogenetic Structure of Alpine Grassland Plant Communities on the Qinghai–Tibetan Plateau with Long-Term Nitrogen Deposition

**DOI:** 10.3390/plants13192809

**Published:** 2024-10-07

**Authors:** Yongqi Liu, Hao Shen, Shikui Dong, Jiannan Xiao, Ran Zhang, Hui Zuo, Yuhao Zhang, Minghao Wu, Fengcai He, Chunhui Ma

**Affiliations:** 1 School of Grassland Science, Beijing Forestry University, Beijing 100083, China; liuyongqi1999@126.com (Y.L.); shenhao2222@gmail.com (H.S.); ran2120921@163.com (R.Z.); abc_zdy@sina.com (H.Z.); 13588741401@163.com (Y.Z.); wuminghao@bjfu.edu.cn (M.W.); fengcai_he0327@163.com (F.H.); luankuaipao@gmail.com (C.M.); 2 School of Environment, State Key Joint Laboratory of Environmental Simulation and Pollution Control, Beijing Normal University, Beijing 100875, China; xjn1009@163.com

**Keywords:** phylogenetic structure, nearest taxon index, net relatedness index, environmental filtration, variation partitioning analysis

## Abstract

Nitrogen (N) deposition rates have notably increased around the world, especially in high-altitude regions like the Qinghai–Tibetan Plateau (QTP). We conducted a six-year comprehensive experiment to simulate nitrogen deposition in an alpine grassland area near Qinghai Lake. Four levels of nitrogen depositions, i.e., 0 (CK), 8 kg N ha^−1^year^−1^ (N1), 40 kg N ha^−1^year^−1^ (N2), and 72 kg N ha^−1^year^−1^ (N3), with three replicates for each N treatment, were tested annually in early May and early July, with the meticulous collection of plant and soil samples during the peak growth period from 15 July to 15 August. We used the null model to evaluate the impact of environmental filtration and interspecific competition on the dynamics of the plant community was assessed based on the level of discrete species affinities within the plant community by constructing a phylogenetic tree. The results showed that the environmental filter was the predominant driver for the change of community’s genealogical fabric. The N2 and N3 treatments increased the influence of soil factors on the change of plant community structure. Climatic factors played a crucial role on the change of plant community in the CK grassland area, while soil factors were dominant in the N1- and N3-treated grasslands.

## 1. Introduction

Nitrogen (N) is known to be an ecologically important factor in a variety of terrestrial ecosystems [1,2,3]. N deposition has increased dramatically over the last century [4,5] and may have an implications for species coexistence in a plant community, favoring nitrophilous plants or non-nitrophilous plants, a classic example of environmental filtration. N deposition on the Qinghai–Tibetan Plateau (QTP) ranged from 8.7 to 13.8 kg N ha−1 year−1 during the 2000s [6] and is predicted to increase to about 40 kg N ha−1 year−1 by 2050 [7]. As the largest ecosystems on the QTP, grasslands have been very sensitive to N deposition [8,9,10,11,12,13,14,15]. In recent decades, N deposition associated with climate change, land use, cover change, and social–economic development has led to grassland degradation and biodiversity loss on the QTP, making the grassland ecosystem more fragile [16,17,18,19,20,21]. Although a large number of researches have been made to examine the effects of mowing, fertilization, and grazing on the species abundance and distribution patterns, functional diversity, and stoichiometry characteristics of the alpine grassland plant community [22,23,24,25,26], few studies have documented the impact of N deposition on the plant community structure and assembly of alpine grasslands on the QTP.

Understanding the processes and mechanisms of mediating the assembly of plant communities has been a long-standing theme in ecology [27,28]. To date, there exist two major prevailing hypotheses on the plant species assemblages of natural communities. The deterministic hypothesis predicts biotic (e.g., competition, facilitation) or abiotic factors (e.g., temperature, precipitation) to be the main forces determining species composition in a community. In contrast, the stochastic hypothesis emphasized that random events (e.g., random dispersal or disturbance events) are the main factors shaping species assemblages [29,30,31,32]. However, the relative importance of deterministic processes and stochastic processes in mediating species assembly in natural communities remained controversial. The deterministic theory suggested that abiotic factors may play an important role in filtering out species without the morphological and/or physiological adaptations to defend against (i.e., establish and reproduce) the existing abiotic conditions in a given area [33]. Environmental filtering has been defined as the effect of environmental gradients or environmental change on the various species in a plant community, favoring some and disfavoring others, resulting in changes of the plant community composition over time [34,35]. For example, families of Caryophyllales have evolved an adaptation strategies order (Aizoaceae, Cactaceae, Didieraceae and Portulacaceae) to survive in arid conditions [36]. According to this hypothesis, it is possible to expect climatic filter favor the co-occurrence of phylogenetically related species, which often share physiological and morphological attributes due to a common evolutionary history [33,37]. However, biotic and abiotic factors could interact simultaneously to determine the final species composition in a community. For instance, variations in climatic conditions can influence the frequency and intensity of species interactions (i.e., competition and facilitation), which in turn influence the final species assemblage in a community [38,39].

Previous researches have focused on the structure and diversity of communities in the dimensions of the environmental conditions and ecological processes (predation, completion, mutualism, etc.) [40]. However, they neglected the fact that historical driver factors have an important influence on the process of community assembly [41,42]. Some scholars have proposed that phylogenetic biology applied to the community ecology [43]. Phylogenetic trees consist of nodes, with nodes representing a taxonomic unit and an evolutionary branch, respectively. The branch’s length is the result of the species’ evolutionary time. The method is based on the phylogenetic tree structure of the plant species within a community and can predict the influence of historical driver factors on an existing community and clarify the main reason for community assembly [33]. To date, botanists and plant ecologists have commonly used the super-tree method to generate phylogenetic hypotheses in their phylogenetic studies of plant communities. In this context, we conducted this study to clarify the properties of community assembly and examine the community structure of alpine grasslands shaped by environmental factors across different gradients of N deposition, answering whether the phylogenetic community structure of alpine grasslands would present cluster dispersion, over dispersion, or random dispersion in response to different levels of N deposition.

## 2. Results

### 2.1. Phylogenetic Tree of Plant Communities

**Figure 1:**
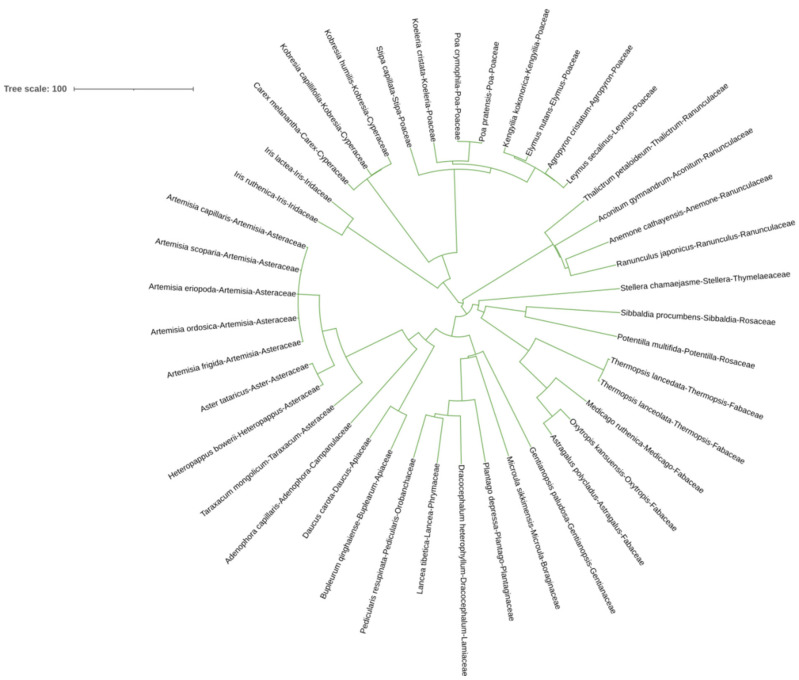
Phylogenetic evolutionary tree of all species in the sample plot.

Figure 1 shows the phylogenetic tree of all species sampled from all the N fertilized plots. Each species presents several divergences in the evolutionary tree. The plant species in each family can be clustered together. There are 16 families and 34 genera of plants in the sampling sites, with a relatively rich species composition and stable community structure.

### 2.2. Changes in the Genealogical Structure of Plant Communities

**Figure 2:**
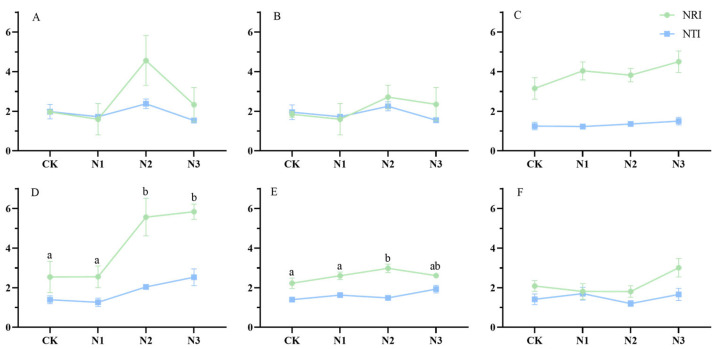
Trajectory of NRI and NTI values of alpine grasslands under different N deposition over six years. Note: (**A**), year 2015; (**B**), year 2016; (**C**), year2017; (**D**), year2018; (**E**), year 2019; (**F**), year 2020. Those with significant differences are labeled by the letters a and b. Results are labeled with lowercase letters to indicate significant differences; those without significant differences are not labeled.

Figure 2 shows the NRI and NTI values consistently surpass zero, indicating that the genealogical structure presents an aggregated configuration under environmental filter. Soil condition changed the community’s genealogical fabric. This determination process predominantly governs the intricacies of community assembly. Generally, the NTI values exhibits a tendency to be smaller than their NRI values, signifying that affinities among end-branch species within the phylogenetic tree exhibit greater dispersion and genealogical distance among all plant communities within different grasslands across N addition gradients. With prolonged N deposition, the spectral structure of the plant community of the alpine grasslands becomes stable. The differences in spectral structure between treatments decrease with N addition year, indicating that the effects of N deposition on phylogenetic structure is declining over time.

### 2.3. Contribution of Soil and Climate Factors in Shaping Community Structure

**Figure 3:**
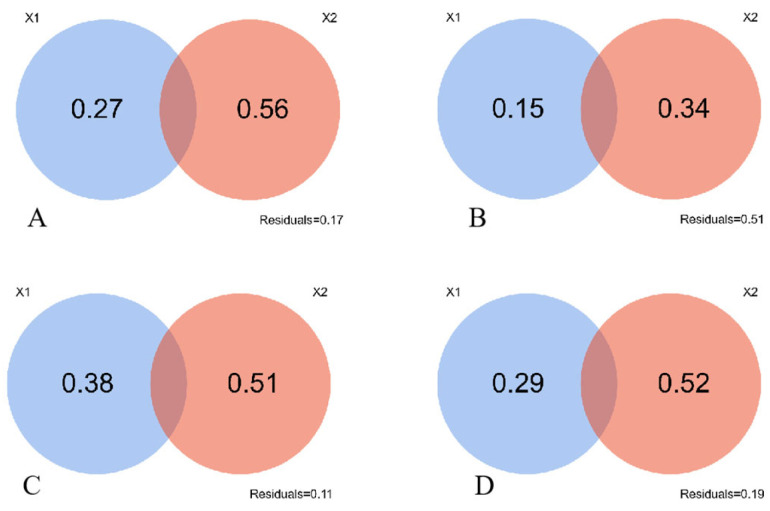
Contribution of soil factors and climatic factors to community structure changes. X1 is the soil factor, X2 is the climate factor. Note: (**A**), CK; (**B**), N1 treatment; (**C**), N2 treatment; (**D**), N3 treatment. The numbers in the figure represent factor contributions.

Figure 3 shows the contribution of soil factors is 0.15 in the N1-treated grasslands, lower than 0.27 observed in the unfertilized grassland (CK). The contributions of soil shaping community structure were 0.38 and 0.29 under N2- and N3-treatments respectively, exhibiting higher values than the control treatment. This implied that N2 and N3 treatments amplify the influence of soil factors to the community structural changes. However, it is worth noting that none of the four treatments exhibit a synthesized effect of both soil factors and climatic factors in changing this community structure.

### 2.4. Correlation of Factors in Shaping Community Structure

**Figure 4:**
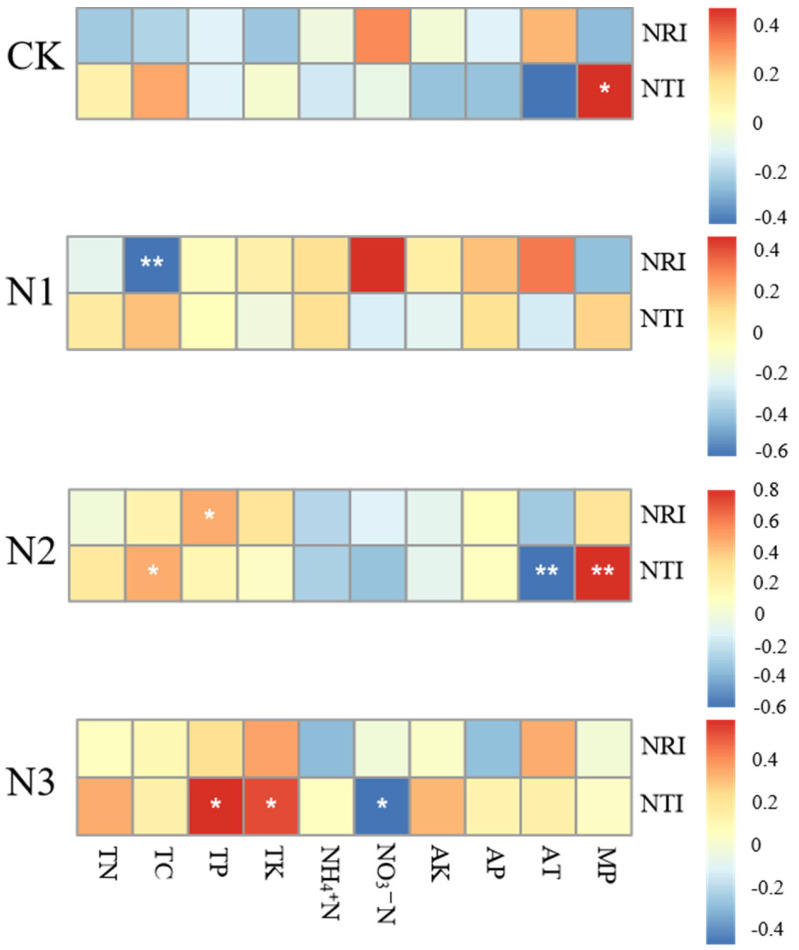
Heatmap of correlation between environmental factors and NRI, NTI. * indicate a significant correlation (*p* < 0.05) and ** indicates a highly significant correlation (*p* < 0.01).

Figure 4 shows the correlations among all environmental factors in shaping the community structure. MP (Monthly precipitation) have a significant positive correlation with NTI under control treatment. In the N1treatment, TC showcases a highly significant negative correlation with NRI. In the N2-treated grassland, TP exhibits a significantly positive correlations with changes in NRI, TC and MP exhibit significantly positive correlations with changes in NTI, while AT (Average monthly temperature) manifests a significantly negative correlation with changes in NTI. In the N3-treated grassland, TP and TK exhibit significantly positive correlations with changes in NTI, while NO3-N displayed a significantly negative correlation with the changes in NTI. All in all, these findings stress the pivotal roles of climatic factors in the control grassland, predominant roles of soil factors in the N1 and N3-treated grasslands, and the joint roles of soil factors and climatic factors in the N2-treated grassland in controlling the spectral structural dynamics. (Detailed soil and climate data can be found in Tables S1–S5).

**Figure 5:**
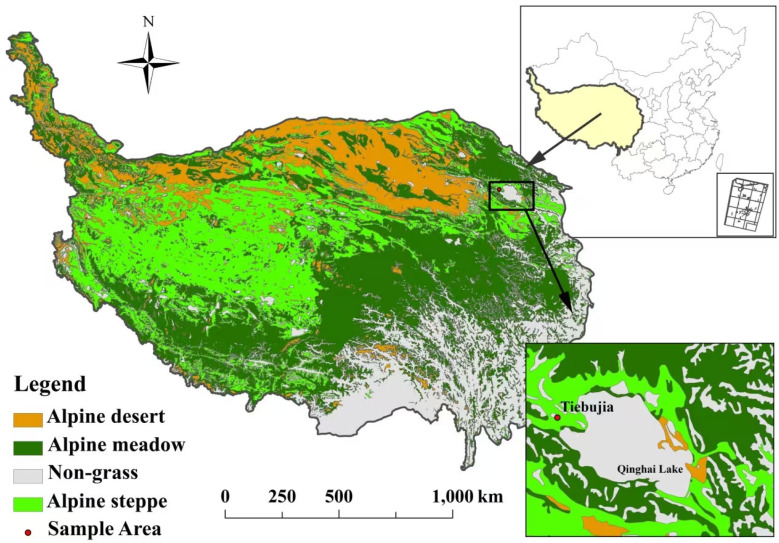
Location of study site.

The heatmap presented in Figure 5 illustrates the correlations between NRI, NTI values, and environmental factors with each treatment.

## 3. Materials and Methods

### 3.1. Study Sites

This study was conducted in Tiebujia Village, Gong he County (99°35′ N, 37°02′ E, 3270 m a.s.l.), which is located at the west sides of Qinghai Lake in Qinghai Province, China. The climate is a typical plateau continental climate with long sunshine hours. The mean annual temperature varies from −0.4 to −1.2 °C, the annual precipitation ranges from 360mm to 430 mm, the annual evaporation is about 1550 mm. The water in the area is clear, low in volume, fast flowing and low in sand content. Precipitation peaks between May and September, with summer precipitation dominating the year [44]. The typical vegetation is the alpine steppe with dominant plant species of *Stipa capillata* and *Poa crymophila*. The soil is predominately sandy loam [45].

### 3.2. Experimental Design

In May 2015, 12 plots (replicates) of 2 m × 5 m were placed in the study site with 1m spacing between plots. The experiment adopted a completely randomized design. The granular ammonium nitrate (NH_4_NO_3_) was randomly added in these plots at 4 levels, i.e., 0 N (CK), 8 kg N ha^−1^year^−1^ (N1), which is equivalent to the annual N deposition on the QTP, 40 kg N ha^−1^year^−1^ (N2), and 72 kg N ha^−1^year^−1^ (N3) with 3 replicates for each N treatment. Nitrogen application experiments by water application. In each treatment, N was applied in two equal doses: the first during the plant rejuvenation period in May, and the second during the growth period in July. The N addition rate of 8 kg N ha^−1^ year^−1^ closely matched the background level of atmospheric N deposition in the area, meaning that the N1 treatment nearly doubled N deposition compared to the control. The N2 treatments represented moderate N deposition, while the N3 treatments simulated high N deposition [46]. Access to climate data through local meteorological monitoring stations.

### 3.3. Vegetation and Soil Sampling

We carried out vegetation surveys, biomass harvests and soil sampling at peak vegetation growth periods in July and early August of each year. Place plant samples in individual envelopes and label them. Plant samples were dried at 60 °C using an oven. Within each of the 12 plots, we sampled all the plant species in randomly placed 1 m×1 m quadrats. Plant sample survey work was carried out following the methods recommended by Ren (1998) [47], recording the names of plant species occurring within the sample plots, as well as their abundance, cover, frequency and height. Meanwhile, the percentage covers of functional groups (grasses, forbs and sedges) were visually estimated. Lastly, we collected three soil cores in each plot using a 3.5 cm-diameter soil probe at a depth of 20 cm. Store and label soil cores in individual self-sealing bags. Soil samples were mixed, air-dried at room temperature to a constant weight, and sieved through a 0.15-mm mesh.

The samples were sealed in polyethylene bags and transported to the lab for extraction. Total nitrogen (TN) and total carbon (TC) were measured using an element analyzer (EA 3000, Langenselbold, Germany). NH_4_^−^-N and NO_3_^−^-N were measured using a flow injection auto-analyzer (AACE, Berlin, Germany). The concentrations of total potassium (TK), total phosphorus (TP), available potassium (AK) and available phosphorus (AP) were measured using inductively coupled plasma spectrometers (ICP; SPECTRO ARCOSEOP, Kleve, Germany).

### 3.4. Data Analysis

The species names were queried through R package ‘plantlist’ and verified whether the species names were APG III phylogenetic acceptance names [48]. Phylogenetic tree files were calculated using R package ‘phylomaker’ and the evolutionary trees were embellished through the iTOL website [49].

Nearest Taxon Index (NTI) and Net Relatedness Index (NRI) were calculated by constructing a null model using R package ‘picante’ [50].

$$NTI=-1\times \frac{MNTDobs-mean(MNTDran)}{sd(MNTDran)}$$

$$NRI=-1\times \frac{MPDobs-mean(MPDran)}{sd(MPDran)}$$

NTI: Nearest Taxon Index; NRI: Net Relatedness Index

MNTD: Mean nearest phylogenetic taxon distance; MPD: Mean phylogenetic distance

obs: survey data; ran: null model generated data

When the NRI and NTI indices were greater than 0, the species in the sample plots had a higher degree of genealogical aggregation than the communities randomly sampled from the species pool, suggesting that the community structure tended to be clustered together by species of similar affinities; when the NRI and NTI indices were less than 0, the species were more dispersed in relation to each other than those randomly sampled from the species pool, suggesting that the community structure tended to be clustered together by species of distant affinities. species were clustered together. The NRI measures the overall similarity of all species, whereas the NTI is more concerned with the effects among the most similar species.

Variation partitioning analysis (VPA) was performed by R package ‘vegan’ for environmental factor partitioning analysis. NTI, NRI and environmental factor correlation were performed through the R package ‘psych’. The correlations between environmental factors and NRI/NTI indices were analyzed by using the R package ‘psych’, while the results were visualized using pheatmap.

The effect of N treatments on both the NTI and NRI was assessed by conducting a one-way ANOVA, with visualized results using Graphpad Prism Software 9. Additionally, the VPA results were visualized via the ‘ggplot2′ package. The evolutionary trees were visualized through the iTOL website.

## 4. Discussion

### 4.1. Responses of Alpine Grassland Plant Community Structure to Nitrogen Deposition

N deposition samples were monitored over a 6-year period. Six years is a better response to changes in plant phylogenetic structure over time scales than shorter-term monitoring. A certain pattern of change can be found through the 6-year monitoring period. Compared with the study of spatial structure, the study of time scales can better reveal the changes in the phylogenetic structure of plant communities [51]. However, the 6-year monitoring period cannot fully reflect the changes in phylogenetic structure over a time scale of many years, and it is still necessary to carry out multi-year monitoring [52].

At the very beginning of N application, the evolutionary tree exhibited consistent patterns; the species numbers remained at 27 in both 2015 and 2016, implying a lower species turnover rate at the initial period of N deposition. However, species richness significantly decreased after three years of continuous N application (Figure S1). In the following years, the species numbers declined the fluctuating trend. In agreement with previous scholars’ findings, we deduce that the declined of species richness is associated with the N deposition [53]. However, species richness did not display a linear reduction but showed a curvilinear decline over time. Despite the absence of a year-by-year decline in species richness, the discernible curvilinear trend accentuates the need to unravel its rationale through subsequent investigations [54].

We deduce that extended N deposition triggered environmental filtering, which may enlarge the changes in plant community structure through analyzing alterations in phylogenetic trees and the shifts in NTI and NRI values. The phylogenetic structure of the plant is influenced by NTI and NRI indices, both of which exceed zero, indicating an aggregation. Thus, it can be inferred that environmental filtration significantly impacts alterations in the plant community structure. The N2 and N4 treatments manifested more pronounced effects on the community’s genealogical structure than the N1 and CK. Notably, the medium-fold N deposition demonstrated the most pronounced impact on genealogical structure in 2015, 2016, and 2019. Conversely, the high ploidy treatment yielded the most conspicuous influence on spectral structure in 2017, 2018, and 2020. Noteworthy distinctions in effects emerge between the N1 and N2 treatments, surpassing those between the N2 and N3 treatments, meaning that medium and high N deposition exerted a more discernible influence on the spectral changes of plant communities than low N deposition [55].

We observed a distinctive pattern of genealogical structure aggregation of the alpine grassland plant community across a gradient of N treatment. This observation underscores the prevailing role of environmental filtering as the key factor shaping the changes in the plant community [56,57]. Notably, the apex of environmental filtering influence was evident in N2-treated grassland in 2015, 2016, and 2019. In the N3-treated grassland, environmental filtering exhibited significant impacts in 2017, 2018, and 2020. The NTI values consistently were smaller than the corresponding NRI values. This discrepancy signifies that species positioned at the terminal branches of the evolutionary tree within the community were characterized by greater genealogical disparity and broader kinship distribution, in contrast to the average genealogical distance [58,59]. Notably, all indices in the grasslands across all gradients of N treatment exceeded 0, signifying that environmental filtering exerted the predominant influence, whereas interspecific competition held a less pronounced role in the dynamics of plant community assembly [60]. Henceforth, we postulate that changes of the genealogical community structure was predominantly driven by the alterations of soil factors associated with the long-term N application. The changes in community structure seem to be chiefly orchestrated by the intricate interplay of environmental filtering factors.

The phylogenetic indices in our study unveiled the conspicuous trend of genealogical community structure of the alpine grassland across different gradients of N application. This finding indicates that environmental filtering played as the vital driver to shape the community structure. The most profound impact of environmental filtering was observed in the alpine grassland under N2 treatment in 2015, 2016, and 2019. Likewise, the effect of environmental filtering was evident in the alpine grassland under N3 treatment in 2017, 2018 and 2020. Moreover, NTI values of the alpine grassland plant community, in comparison to NRI, exhibited significant decline with the increase of N deposition. This disparity implies that species situated at the termini of the evolutionary tree showcased greater genealogical remoteness and broader kinship dispersion when compared to the mean genealogical distances [61]. In all the grasslands under different N application treatments, all the indices exceeded 0. This fact underscores the preeminence of environmental filtering as the principal driver shaping the community structure, relegating interspecific competition to a secondary driver in the process of community assembly [62,63]. Hence, it is clear that the changes in the genealogical community structure were primarily driven by the alterations in soil factors associated with long-term N application. In agreement with previous scholars’ findings [64,65], we believe the changes in community structure were predominantly governed by environmental filtering.

### 4.2. Role of Soil Factors Shaping Change Patterns of Phylogenetic Structure

A discernible pattern in the changes of the spectral structure of the plant community across N application gradients was detected through the meticulous application of VPA analysis. Importantly, none of the meticulous VIF variance inflation factors exceeded 10, thus adhering to the prerequisites for conducting VPA analysis. Notably, the impacts of soil factors on the changes in the spectral structure of the plant community were quite low at the low N addition gradient (N1), but they showed marked effects on the changes in the spectral structure of the plant community at the medium N addition gradient (N2) and high N addition gradient (N3). In agreement with previous scholars’ findings [35], a noteworthy observation pertained to the absence of a combined effect of climate factors and soil factors under N addition treatments. Among all the alpine grasslands treated with different gradients of N addition, the most substantial contribution of soil factors was identified under the N2 treatment.

We found that TC, TP, TK, and NO_3_-N were the most pronounced among all the soil factors, and the role of soil factors was increasingly intensified along the N application gradients. Our previous investigation identified that TP was a pivotal factor to shape the plant community structure [66]. Apart from TP, we found in the present study that TC, TK, and NO_3_-N were influential factors for the plant community assembly under long-term N deposition. The previous study revealed that N application directly changed soil N content, resulting in a shift of soil C: N and N: P ratios, the most important stoichiometry for plant growth [67]. The changes of C: N, and N: P may lead to changes in plant community assembly. The change of soil TK with N deposition can also affect plant growth and community assembly. In this study, we found that TP, TK, and MP were significantly positively correlated with phylogenetic structure, while NO_3_-N and AT were significantly negatively correlated with phylogenetic structure under all N treatments. TC was negatively correlated with phylogenetic structure under N1 treatment, while it was positively correlated with phylogenetic structure under N3 treatment. Notably, these alterations have been accompanied by discernible changes in the spectral structure, culminating in a marked tendency towards aggregation.

## 5. Conclusions

Our findings shed light on the nuanced impacts of future N deposition on plant community dynamics of the alpine grassland. It can be generally concluded that modest N deposition dampens the influence of environmental filtration in plant community assembly, while elevated N deposition may amplify the role of environmental filtration. Low N deposition curtails the significance of soil factors, whereas high N deposition accentuates the prominence of soil factors. Over six years of observation, a discernible trend towards stabilization becomes evident in the NRI and NTI values. Simultaneously, the prevalence of environmental filtration gradually wanes in its role in shaping plant community structure. It is noteworthy that both NRI and NTI values consistently exceed zero, affirming the preeminence of environmental filtration in the plant community’s structural genesis. Specifically, NRI are a valuable tool for visualizing the trajectory of the entire community’s genealogical structure, can illuminate the key actors driving environmental filtering. In contrast to NRI, NTI exhibits relatively fewer fluctuations throughout the period of continuous N application. This finding implied that minimal variability exists among the closest kin within the plant community structure. In summary, our study highlights insights into the interplay between N application, environmental filtration, and community structure dynamics, thereby contributing to the broader understanding of responses of the alpine grassland plant community to N deposition.

## Data Availability

All the data provided in this study are available within this article.

## Supplementary Materials

The following supporting information can be downloaded at: https://www.mdpi.com/article/10.3390/plants13192809/s1, Table S1: Soil nutrients data for each year in the CK treatment; Table S2: Soil nutrients data for each year in the N1 treatment; Table S3: Soil nutrients data for each year in the N2 treatment; Table S4: Soil nutrients data for each year in the N3 treatment; Table S5: Average Monthly Temperature (AT) and Monthly Precipitation (MP) Data from July 15 to August 15 of each year; Figure S1: The number of plant species in the plot
