# Supplementary material for: Changes in the Phylogenetic Structure of Alpine Grassland Plant Communities on the Qinghai–Tibetan Plateau with Long-Term Nitrogen Deposition
Source: Plants (Basel). 2024 Oct 7;13(19):2809. doi: 10.3390/plants13192809 (PMC11479209; doi:10.3390/plants13192809)

Table S1: Soil nutrients data for each year in the CK treatment

Note: TN: Total Nitrogen, TC: Total Carbon, TP: Total Phosphorus, TK: Total Potassium, AP: Available Phosphorus, AK: Available Potassium

year	TN(%)	TC(%)	TP(ug/g)	TK(ug/g)	NH4-N(mg/L)	NO3-N(mg/L)	AK(ug/mL)	AP(ug/mL)
	0.196	3.788	650	19292	0.455	2.703	14.91	0.063
2015	0.215	3.615	588	18924	0.163	1.698	11.84	0.052
	0.23	3.918	629	19385	0.342	4.12	19.64	0.043
	0.33	4.177	660.345	20230	1.365	4.157	30.473	0.135
2016	0.34	4.16	629.351	19583.5	1.252	4.165	23.952	0.127
	0.32	3.983	666.531	20265.9	0.922	4.066	19.98	0.138
	0.248	3.424	633.318	19404.167	0.995	6.956	17.363	0.086
2017	0.177	3.059	600.274	18776.367	0.892	8.667	12.189	0.026
	0.209	3.011	634.768	19498	1.023	7.769	16.822	0.144
	0.364	4.301	709.333	20120	0.15	0.8	69.767	0.282
2018	0.331	3.969	729	20717.333	0.15	0.87	33.733	0.167
	0.321	4.263	702.667	20642	0.19	0.5	25.933	0.152
	0.276	3.223	707.333	20641	4.213	3.123	44	0.211
2019	0.219	3.129	726.333	21308.333	4.054	2.578	38.333	0.279
	0.222	3.134	707	20267	2.532	1.85	24.1	0.197
	0.3	3.987	635.333	19153.333	0.588	2.793	20.563	0.12
2020	0.293	3.863	627	19342	0.221	2.725	15.67	0.133
	0.31	3.883	631.667	20071.333	0.798	3.47	18.937	0.058

Table S2: Soil nutrients data for each year in the N1 treatment

year	TN(%)	TC(%)	TP(ug/g)	TK(ug/g)	NH4-N(mg/L)	NO3-N(mg/L)	AK(ug/mL)	AP(ug/mL)
	0.225	3.812	679	19723	0.27	3.708	11.61	0.052
2015	0.273	3.632	656	19291	0.172	4.421	10.12	0.114
	0.277	3.714	626	20094	0.115	4.131	11.78	0.074
	4.163	0.366	703.805	20646.5	1.204	4.135	23.067	0.088
2016	4.411	0.346	633.211	18967.6	1.488	4.06	29.77	0.116
	4.194	0.354	672.252	19929.6	1.136	4.188	20.799	0.166
	0.261	3.23	628	19029	0.747	10.683	20	0.057
2017	0.249	3.434	607	19548	0.825	11.44	24.1	0.128
	0.227	3.255	622	19194	0.903	10.2	16.7	0.052
	0.351	4.208	719.3	20706	0.15	0.6	17.8	0.05
2018	0.332	4.047	727.7	21232	0.16	0.56	26.2	0.071
	0.328	3.994	700	20478	0.23	0.7	18.64	0.08
	0.243	3.124	726.333	20665.333	3.913	3.212	22.167	0.094
2019	0.207	3.306	734	21374.333	3.651	2.953	28.667	0.110
	0.242	3.325	731.667	20374.667	3.304	2.522	27.367	0.084
	0.31	3.63	613	19391	0.559	2.735	18.9	0.077
2020	0.31	4.07	606	18413	0.232	2.75	18.13	0.029
	0.29	3.96	622	19459	0.742	4.186	20.36	0.049

Table S3: Soil nutrients data for each year in the N2 treatment

year	TN(%)	TC(%)	TP(ug/g)	TK(ug/g)	NH4-N(mg/L)	NO3-N(mg/L)	AK(ug/mL)	AP(ug/mL)
	0.275	3.708	640	19860	0.102	3.874	22.79	0.084
2015	0.239	3.717	656	19567	0.106	2.975	10.67	0.091
	0.281	3.762	667	19319	0.042	2.417	6.96	0.051
	0.352	4.277	657.506	19755.5	1.225	4.351	20.622	0.122
2016	0.312	4.086	626.423	20231.5	1.254	4.327	20.248	0.13
	0.336	4.106	639.441	19344.8	1.032	4.405	24.191	0.175
	0.192	2.995	606.129	18386	0.852	9.946	18.127	0.027
2017	0.313	3.84	629.423	19808.667	0.87	10.551	16.832	0.185
	0.216	3.151	635.31	19749.033	0.999	9.838	14.716	0.087
	0.311	4.263	728.333	20215	0.16	0.77	24.4	0.139
2018	0.334	4.121	689.333	20423.333	0.15	0.76	20.633	0.107
	0.346	4.259	741	20805.333	0.15	0.82	21.133	0.107
	0.259	3.111	721.667	20143	5.004	2.107	27.333	0.16
2019	0.299	3.463	699	20322	4.452	2.073	22.8	0.107
	0.306	3.175	743.333	20679.667	3.726	3.28	17.667	0.139
	0.297	3.817	628	19707.333	0.229	2.891	14.92	0.136
2020	0.283	3.86	621.667	19783.667	0.657	4.709	21.08	0.057
	0.313	3.95	622	19059.667	0.233	2.82	14.487	0.048

Table S4: Soil nutrients data for each year in the N3 treatment

year	TN(%)	TC(%)	TP(ug/g)	TK(ug/g)	NH4-N(mg/L)	NO3-N(mg/L)	AK(ug/mL)	AP(ug/mL)
	0.264	3.6	656	19291	1.04	4.4	10.1	0.162
2015	0.269	3.7	661	19318	1.12	2.9	8	0.12
	0.262	3.6	637	20084	0.802	3.6	12.4	0.108
	0.297	3.759	661	19734	1.1	4.4	23.5	0.13
2016	0.335	4.059	640	20157	1.4	4.5	20	0.13
	0.332	4.01	639	19493	1.2	4.4	20.2	0.14
	0.296	3.699	618.615	19975	0.643	11.401	12.216	0.028
2017	0.195	2.967	613.147	19349.867	0.246	8.002	13.948	0.019
	0.325	3.907	617.673	19759.8	0.691	11.723	14.630	0.025
	0.33	3.9	728	20811	0.1	1	24	0.16
2018	0.36	4.2	752	20760	0.2	1.1	22.5	0.12
	0.33	3.9	743	21042	0.2	1.1	17	0.1
	0.28	3.9	730	20458	5.8	2.4	27.9	0.16
2019	0.3	3.5	744	20751	5.9	3.3	24.4	0.14
	0.28	3.8	750	20978	6.2	3.6	18.9	0.08
	0.30	3.8	638	19791	0.8	2.8	15.5	0.14
2020	0.35	4.2	578	19194	0.5	2.8	14.1	0.08
	0.29	3.9	615	19445	0.2	2.8	22.8	0.1

Table S5:Average Monthly Temperature (AT) and Monthly Precipitation (MP) Data from July 15 to August 15 of each year

year	AT(℃)	MP(mL)
2015	10.4	99.8
2016	12.4	103.7
2017	12.7	86.5
2018	12.4	98.8
2019	10.6	88.3
2020	11.2	113

Figure S1:The number of plant species in the plot

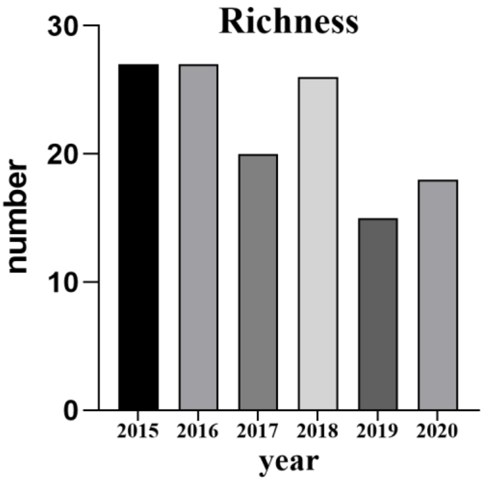

Supplement: Supplementary file 1 [file plants-13-02809-s001.zip › plants-3142572-supplementary.pdf]
